# A novel siderophore system is essential for the growth of *Pseudomonas aeruginosa* in airway mucus

**DOI:** 10.1038/srep14644

**Published:** 2015-10-08

**Authors:** Mia Gi, Kang-Mu Lee, Sang Cheol Kim, Joo-Heon Yoon, Sang Sun Yoon, Jae Young Choi

**Affiliations:** 1 Department of Otorhinolaryngology, Brain Korea 21 PLUS Project for Medical Science, Seoul, Korea; 2 Department of Microbiology and Immunology, Brain Korea 21 PLUS Project for Medical Science, Seoul, Korea; 3 Institute for Immunology and Immunological Diseases, Yonsei University College of Medicine, Seoul, Korea

## Abstract

*Pseudomonas aeruginosa* establishes airway infections in Cystic Fibrosis patients. Here, we investigate the molecular interactions between *P. aeruginosa* and airway mucus secretions (AMS) derived from the primary cultures of normal human tracheal epithelial (NHTE) cells. PAO1, a prototype strain of *P. aeruginosa*, was capable of proliferating during incubation with AMS, while all other tested bacterial species perished. A PAO1 mutant lacking *PA4834* gene became susceptible to AMS treatment. The Δ*PA4834* mutant was grown in AMS supplemented with 100 μM ferric iron, suggesting that the *PA4834* gene product is involved in iron metabolism. Consistently, intracellular iron content was decreased in the mutant, but not in PAO1 after the AMS treatment. Importantly, a PAO1 mutant unable to produce both pyoverdine and pyochelin remained viable, suggesting that these two major siderophore molecules are dispensable for maintaining viability during incubation with AMS. The Δ*PA4834* mutant was regrown in AMS amended with 100 μM nicotianamine, a phytosiderophore whose production is predicted to be mediated by the *PA4836* gene. Infectivity of the Δ*PA4834* mutant was also significantly compromised *in vivo*. Together, our results identify a genetic element encoding a novel iron acquisition system that plays a previously undiscovered role in *P. aeruginosa* airway infection.

*Pseudomonas aeruginosa* is a highly adaptable Gram-negative bacterium that colonizes various environmental niches and causes major airway infections. Notably, 60–70% of patients with cystic fibrosis (CF) are infected by *P. aeruginosa* in the airway as the disease progresses to the age of 20^1^. As a major opportunistic pathogen, *P. aeruginosa* also infects patients suffering from ventilator-associated pneumonia^2^ or burn wounds^3^. Previous studies demonstrated that thickened airway mucus caused by mutations in the cystic fibrosis transmembrane conductance regulator (*CFTR*) gene may be less effective in eliminating microbial invaders^4^ due in part to decreased mucociliary clearance^5^. The concentrations of major ions in the airway mucus have been precisely determined and were not significantly different between normal and CF samples^6^, supporting the notion that isotonic volume changes in airway surface liquid accounts for defective mucociliary clearance followed by *P. aeruginosa* infection in the CF airway^7^. Furthermore, the abnormally altered CF airway was found to be anaerobic^8^ and *P. aeruginosa* was found to form robust biofilms during anaerobiosis^9^,^10^,^11^. However, these findings do not fully explain why *P. aeruginosa* has been exceptionally capable of establishing chronic airway infections.

Airway mucus contains various antibacterial components such as lysozyme^12^, lactoferrin^12^ and IgA^13^, which suppress bacterial growth on the airway surface. Notably, elevated lysozyme activity and lactoferrin levels were observed in the bronchoalveolar lavage fluid (BALF) derived from CF patients^14^. In the same study, it was also shown that lysozyme and lactoferrin levels were increased in older CF patients^14^. These data suggest that the degree of *P. aeruginosa* infection may not correlate with the levels of these molecules in the CF airway and frequent *P. aeruginosa* infection is likely ascribed to its ability to effectively respond to host-specific hostile environments.

Iron is essential for bacterial survival and typical bacterial organisms require micromolar levels of iron for optimal growth^15^,^16^. However, the utilization of iron is limited by the host as most iron is bound to circulating proteins such as transferrin, lactoferrin, and ferritin *in vivo*^17^. To sequester iron for their own use, bacterial species possess a myriad of mechanisms that regulate the expression, secretion, and internalization of iron chelating compounds termed siderophores. Bacterial iron metabolism has been extensively studied using *P. aeruginosa* as a model organism. Pyochelin and pyoverdine are well-characterized siderophore molecules that *P. aeruginosa* produces under iron-limited conditions^18^. Siderophore-mediated processes also participate in virulence regulation of *P. aeruginosa*^19^,^20^. Intriguingly, pyoverdine-defective *P. aeruginosa* strains have been detected in CF sputa^21^,^22^. Moreover, a PAO1 mutant defective in both pyochelin and pyoverdine was found to colonize the lungs of immunocompromised mice, even though its virulence was attenuated^23^. These results indicate that additional iron-acquisition mechanisms may play a more important role during airway infection. In support of this notion, diverse iron acquisition pathways have been reported in *P. aeruginosa*, including heme uptake systems^24^, TonB-dependent uptake systems^25^, and the FeOABC system^26^.

Airway mucus is a frontline defense of the innate immune response against invading bacterial pathogens; however, physiological changes induced in *P. aeruginosa* during interactions with airway mucus are not clearly understood at the molecular genetic level. In this study, we investigated various bacterial responses to airway mucus secretions (AMS) harvested from primary cultures of normal human tracheal epithelial (NHTE) cells. Unlike other bacterial species of clinical significance, *P. aeruginosa* exhibited resistance to treatment with AMS and was capable of replicating in its presence as well. We took a genome-wide approach to uncover a genetic determinant responsible for a previously uncharacterized iron uptake mechanism. This report provides novel insight into the interaction between *P. aeruginosa* and the host, especially at the early stages of airway infection. In addition, this work proposes a drug target, the inhibition of which may contribute to the efficient eradication of this important pathogen.

## Results

### *P. aeruginosa* exhibits exceptional resistance in response to incubation with airway mucus secretions (AMS)

**Figure 1:**
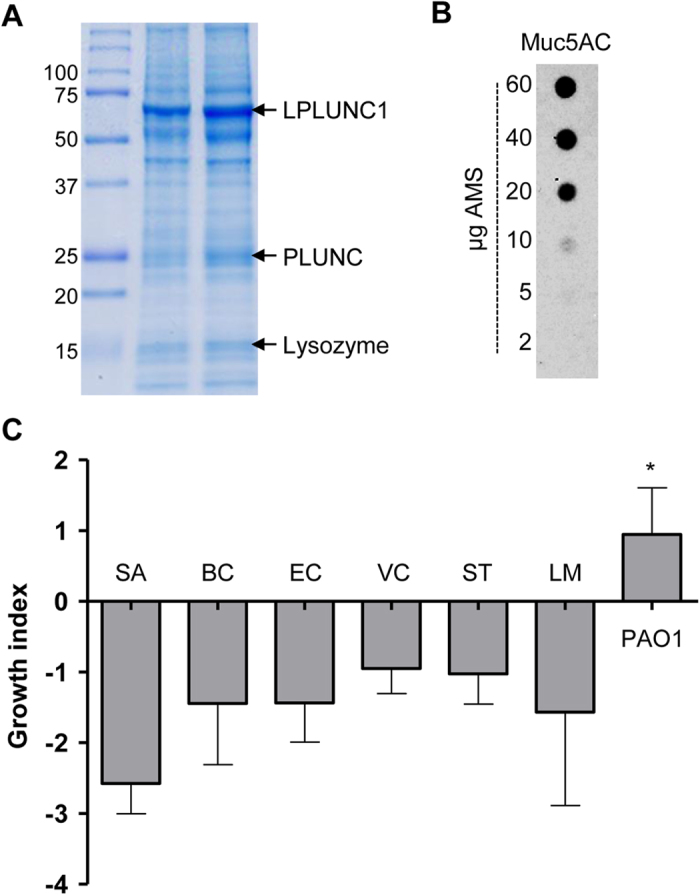
Preparation of AMS and robust ability of *P. aeruginosa* to grow in AMS. (**A**) SDS-PAGE analysis of two independently prepared AMS samples. Ten μg of each sample was loaded into 12% SDS-PAGE and three distinct bands were identified by Mass Spectrometry. (**B**) Dot-blot analysis of Muc5AC protein. AMS samples with indicated protein amount were immobilized in the nitrocellulose membrane and probed with anti-Muc5AC antibody. (**C**) Seven different bacterial strains (SA, *Staphylococcus aureus*; BC, *Bacillus cereus*; EC, *Escherichia coli*; VC, *Vibrio cholerae*; ST, *Salmonella enterica* serovar *Typhimurium*; LM, *Listeria monocytogenes*; and PAO1, *P. aeruginosa*) were incubated with AMS prepared from primary cultures of normal human tracheal epithelial cells for 16 h. Changes in bacterial cell viability were monitored by calculating the growth index as described in the Materials and Methods. Three independent experiments were performed, and the mean values ± SD (error bars) are displayed in each bar. *p < 0.05 vs. other species, as determined by ANOVA.

Airway mucus contains a variety of antimicrobial agents^27^ serving as a frontline immune defense against invading microorganisms. We first examined whether our primary culture system produced secretions similar to those found in the human airway. To address this issue, we analyzed protein components of the AMS recovered from the differentiated NHTE cells. The SDS-PAGE shown in Fig. 1A indicates that previously characterized proteins, such as LPLUNC1^28^, PLUNC^29^, and lysozyme^30^ were detected in our two independent AMS samples. Mucin, a highly glycosylated protein, is too big to be separated in our SDS-PAGE. Our dot blot analysis, however, using an anti-Muc5AC antibody, clearly demonstrated the presence of the mucin in large quantity (Fig. 1B). We used anti-Muc5AC antibody for detecting mucin, because Muc5AC is a major component of the airway mucins^31^. We then examined how AMS exerts its effect on various bacterial species including human pathogens by comparing the number of viable cells before and after incubation with AMS. Bacterial growth was monitored by measuring the growth index as described in the Methods section. After 16 h incubation with AMS, all of the tested bacterial species, except for *P. aeruginosa* lost their viability. *S. aureus* was the most susceptible to the treatment with AMS. In contrast, the growth index of PAO1, the prototype *P. aeruginosa* strain, was increased after the same treatment (Fig. 1C).

### Genome-wide expression analysis followed by mutant screening identified a PAO1 mutant susceptible to AMS treatment

Our results show that PAO1 is exclusively resistant to the antimicrobial action of AMS, which *P. aeruginosa* cells likely encounter during the early stages of the airway infection. To offer clues into the molecular basis of this unique resistance, we performed a microarray analysis. Table 1 shows a list of the top 35 genes that were most upregulated during incubation with AMS vs. PBS. Exposure to AMS influenced bacterial transcription dramatically, as evidenced by the extent to which expression was changed (~7- to ~56-fold increase). A substantial number of genes (16 genes) were determined to encode hypothetical proteins. Of note, four genes (*pchB*, *pchG*, *pchE*, and *pchF*) involved in the biosynthesis of pyochelin, a siderophore molecule for iron acquisition^32^, were included in the list. Furthermore, expression of the *pvdS* gene encoding a sigma factor that activates the production of pyoverdine, another iron chelator^33^, was also highly induced in PAO1 cells grown in the presence of AMS. Transcript levels of two genes (*PA4834* and *PA4837*) constituting an operon were also highly induced in response to AMS treatment (Table 1).

**Figure 2:**
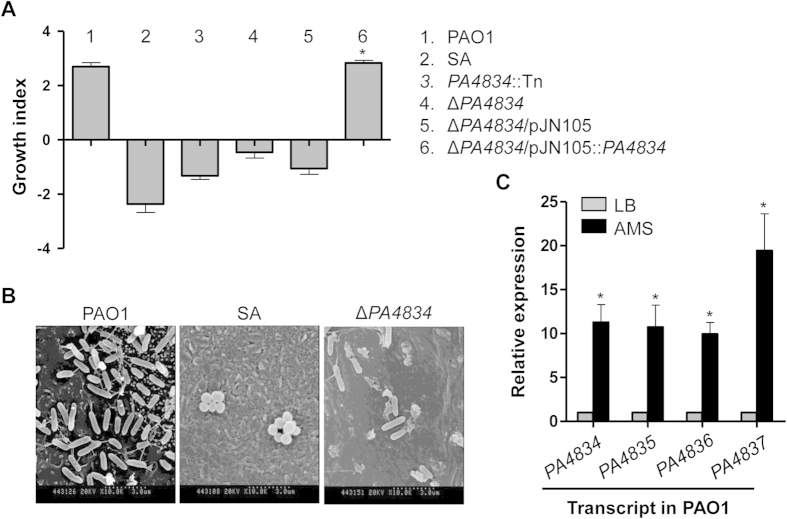
A PAO1 mutant devoid of the *PA4834* gene lost its ability to grow in AMS. (**A**) Bacterial strains indicated by numbers were grown in AMS, and the growth index for each strain was measured. Experimental conditions were identical to those described in Fig. 1C. Six independent experiments were performed, and mean values ± SD (error bars) are displayed in each bar. SA, *S. aureus*. *p < 0.05 vs. growth indices of *PA4834*::Tn, Δ*PA4834*, and Δ*PA4834*/pJN105, as determined by students’ t-test. (**B**) Scanning electron microscope of primary cultured NHTE cells infected with PAO1, SA and Δ*PA4834* mutant. The images were acquired at a magnification of 10,000. (**C**) Quantitative real-time PCR was performed to assess *PA4834*, *PA4835*, *PA4836*, and *PA4837* gene transcript levels. PAO1 cells were grown in LB (gray bars) or AMS (black bars) before RNA extraction. Transcript levels of the tested genes indicated at the bottom were normalized with those of the *rpoD* gene transcript. Three independent experiments were performed, and mean values ± SD are displayed in each bar. *p < 0.05 vs. expression levels of each gene in LB-grown PAO1, as determined by students’ t-test.

Next, we assessed the effect of disruption of each gene on the susceptibility of PAO1 to AMS treatment. Transposon (Tn) insertion mutants were grown in AMS and the growth index of each mutant was calculated (Table 2). All of the mutants grew fine in LB media (data not shown). While the 31 tested mutants exhibited varying degrees of growth, most of them showed positive growth index values (Table 2). Of importance is that mutations in either *PA4063* or *PA4834* resulted in growth inhibition in our initial screen (growth index <0). Subsequent experiments indicated that the *PA4834* Tn mutant was consistently susceptible to treatment with AMS. To further validate this result, we constructed an in-frame deletion mutant of *PA4834* and the mutant also lost its viability when treated with AMS (growth index of approximately −1.0), although the degree of viability loss was not as significant as what was observed in the *S. aureus* strain (Fig. 2A). Importantly, restored ability to grow in AMS was observed when the mutant was complemented with a plasmid-born wild-type copy of the *PA4834* gene (Fig. 2A). The Δ*PA4834* mutant grew completely fine in LB (data not shown) and was equally sensitive to a range of antibiotics with its parental strain, PAO1 (Table S4). These results suggest that the overall growth-associated phenotypes were not affected much by the *PA4834* gene disruption.

For investigation of bacterial colonization in NHTE cells, primary NHTE cells were differentiated at an air–liquid interface (ALI) for 8 days. PAO1, SA and Δ*PA4834* mutant was inoculated into the apical region of the NHTE cells and incubated for 14 h. Figure 2B represents scanning electron microscope (SEM) images of the infected NHTE cells with bacterial cells. PAO1 invaded the NHTE cells and was proliferated (Fig. 2B). In contrast, only a few numbers of SA and Δ*PA4834* mutant remained and the viability of NHTE cells was not affected (Fig. 2B). Quantitative RT-PCR analysis showed that PAO1 has an 11-fold increased level of *PA4834* gene transcript when grown in AMS vs. LB (Fig. 2C), further suggesting its key role during the interaction with AMS. In addition, *PA4835*, *PA4836* and *PA4837* genes were also highly transcribed in response to the treatment with AMS (Fig. 2C).

*PA4834* is a component of a four-gene operon (*PA4834*-*PA4837*) and is predicted to encode a membrane-associated protein (www.pseudomonas.com). The *PA4836* and *PA4837* genes encode proteins homologous to nicotianamine synthase and a TonB-dependent siderophore receptor, respectively (Table S3). Table S3 shows a list of bacterial species that were found to possess genes homologous to each component of the operon. *P. aeruginosa* strains contain all four genes, each with >99% sequence identity to the corresponding gene in PAO1. Sequence identities between cognate genes in PAO1 vs. PA7 were somewhat lower (91~96%) at the nucleotide level (Table S3). *Serratia* sp. FS14 was found to harbor two genes similar to *PA4834* and *PA4835*, with higher identity to the *PA4834* gene (Table S3). It is of interest that these two genes also constitute a likely six-gene operon, predicted to be involved in iron metabolism (data not shown). Other genomes on the list contain only one gene homologous to a corresponding gene of the *PA4834-PA4837* operon (Table S3). Of note, the bacterial species tested in Fig. 1C were determined not to possess genetic elements similar to any part of the *PA4834-PA4837* operon. Together, these results suggest that the *PA4834-PA4837* operon is exclusively found in *P. aeruginosa*, and not in other bacterial species with sequenced genomes.

### The *PA4834* gene product is essential for iron acquisition during the interaction with AMS

Our microarray analysis indicated that genes involved in iron metabolism were highly transcribed in response to AMS treatment. Moreover, *PA2033*, annotated as a hypothetical gene, is predicted to encode a protein homologous to a siderophore-interacting protein (www.pseudomonas.com). All of this information led us to hypothesize that PAO1 likely activates its response to acquire iron during treatment with AMS, and *PA4834* gene deficiency may result in the failure of such a process. In line with this notion, AMS was reported to contain large amounts of lactoferrin^12^,^34^ and transferrin^35^, which can sequester free iron to restrict bacterial growth.

**Figure 3:**
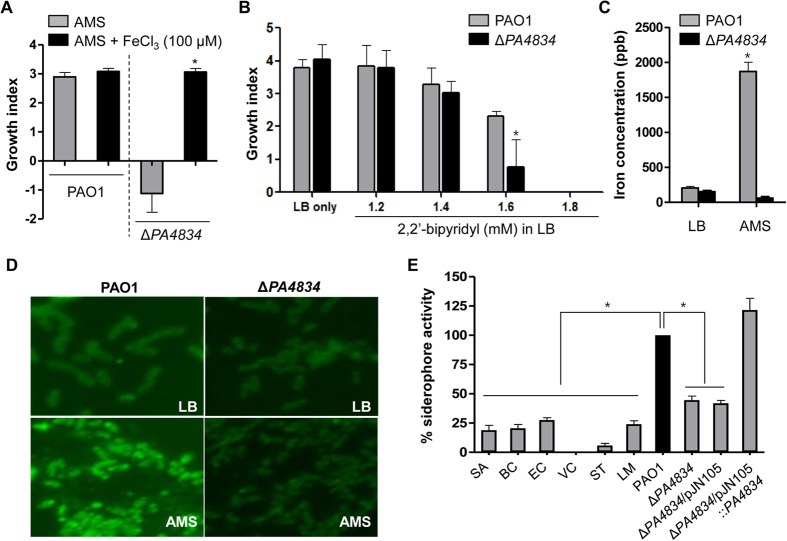
The *PA4834* gene product plays a role in iron acquisition. (**A**) Effect of ferric iron on growth of the Δ*PA4834* mutant in AMS. Growth indices of PAO1 and the Δ*PA4834* mutant were measured after growth in AMS without (grays bars) or with (black bars) 100 μM FeCl_3_. Six independent experiments were performed, and mean values ± SD (error bars) are displayed in each bar. *p < 0.05 (students’ t-test) vs. growth of the Δ*PA4834* mutant in AMS without FeCl_3_. (**B**) Effect of an iron chelator on bacterial growth. Growth indices of PAO1 (gray bars) and the Δ*PA4834* mutant (black bars) were calculated after growth in LB supplemented with increasing concentrations of 2, 2′-bipyridyl. Bacterial cells were grown for 12 h. Three independent experiments were performed, and mean values ± SD (error bars) are displayed in each bar. *p < 0.05 (students’ t-test) vs. PAO1 growth in the same media. (**C**) Intracellular iron concentration of PAO1 (gray bars) and the Δ*PA4834* mutant (black bars) after growth in LB or in AMS. Inductively coupled plasma mass spectrometry (ICP-MS) was used for the measurement and iron concentrations are displayed in parts per billion (ppb). Three independent experiments were performed, and mean values ± SD (error bars) are displayed in each bar. *p < 0.05 (students’ t-test) vs. all other values. (**D**) Fluorescent microscope images of PAO1 and the Δ*PA4834* mutant. After growth in LB or AMS, bacterial suspensions were stained with PhenGreen™ SK, diacetate. Experiments were repeated three times and representative images, processed at the same magnification, are shown. **(E)** Bacterial strains indicated at the bottom were grown in LB for 16 h and cell-free culture supernatants were subject to the iron sequestration assay. The iron sequestration activities of indicated species were normalized with that of PAO1 (black bar). SA, *Staphylococcus aureus*; BC, *Bacillus cereus*; EC, *Escherichia coli*; VC, *Vibrio cholerae*; ST, *Salmonella enterica* serovar *Typhimurium*; LM, *Listeria monocytogenes*. Three independent experiments were performed, and mean values ± SD (error bars) are displayed in each bar. *p < 0.05 (students’ t-test) vs. all other strains but Δ*PA4834*/pJN105::*PA4834*.

When the Δ*PA4834* mutant was grown in AMS supplemented with 100 μM ferric iron, its viability was completely recovered (Fig. 3A). The growth index of wild-type PAO1 was comparable in the presence or absence of added FeCl_3_. We then compared the relative sensitivity of PAO1 and the mutant under iron-depleted conditions. PAO1 showed better resistance to the presence of increasing concentrations of 2,2′-bipyridyl, an iron chelator. At a 1.6 mM concentration, PAO1 grew significantly better than the Δ*PA4834* mutant (Fig. 3B). ICP-MS analysis is a useful technique to measure total iron concentration in bacterial cells^36^. Intracellular iron concentration of the mutant was only slightly lower than PAO1, when strains were grown in LB (Fig. 3C). However, a dramatic difference was induced when the strains were grown in AMS. While a significantly increased level of intracellular iron was detected in AMS-grown PAO1, iron concentration in the Δ*PA4834* mutant was decreased under the same growth condition (Fig. 3C). This result suggests that PAO1 cells, but not the mutant cells, are capable of activating cellular machineries to uptake iron. Likewise, when bacterial cells were stained with PhenGreen™ SK, diacetate, a green-fluorescent heavy metal indicator, a sharp increase in green fluorescent signal was detected in PAO1 grown in AMS vs. LB (Fig. 3D). Consistent with the ICP-MS results, such an increase was not observed in the Δ*PA4834* mutant, further demonstrating that possession of an intact *PA4834* gene is crucial for adjusting bacterial physiological status to an adverse environment, where iron limitation is involved.

To compare the relative siderophore activities of bacterial species, the iron sequestration assay was performed using SideroTech Kit (Fig. 3E). The result demonstrated that (i) PAO1 produced by far the largest amount of siderophore and (ii) all other species found to be susceptible to the AMS treatment (Fig. 1C) produced significantly low levels of siderophore. This result further suggests that resistant growth of *P. aeruginosa* in AMS is strongly related with its robust iron sequestration activity. Importantly, iron sequestration activity was ~2-fold decreased in the Δ*PA4834* mutant, which was completely restored by *PA4834* gene complementation.

### Pyochelin and pyoverdine are dispensable for maintaining viability during incubation with AMS

**Figure 4:**
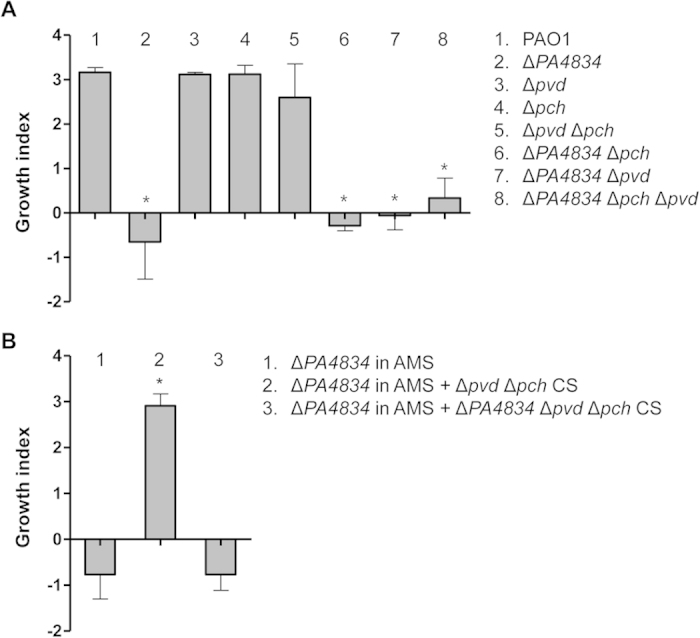
Growth of PAO1 pyochelin and pyoverdine mutants in AMS. (**A**) Bacterial strains indicated by numbers were grown in AMS for 16 h and the growth index of each strain was measured. Experimental conditions were identical to those described in Fig. 1C. Three independent experiments were performed, and mean values ± SD (error bars) are displayed in each bar. *p < 0.05 vs. growth indices of PAO1, Δ*pvd*, Δ*pch*, and Δ*pvd*Δ*pch*, as determined by students’ t-test. (**B**) Effect of bacterial culture supernatant (CS) on Δ*PA4834* mutant growth in AMS. The Δ*PA4834* mutant was grown in AMS supplemented with the CS from the Δ*pvd*Δ*pch* double or *ΔPA4834ΔpvdΔpch* triple mutant. CSs were obtained from bacterial cultures grown in LB and diluted 10-fold in AMS. Three independent experiments were performed, and mean values ± SD (error bars) are displayed in each bar. *p < 0.05 vs. growth of the Δ*PA4834* mutant in AMS or AMS amended with the CS from the triple mutant, as determined by students’ t-test.

Our results demonstrate that expression of *PA4834* is substantially stimulated upon exposure to AMS, which likely encodes a protein critical for iron acquisition. In *P. aeruginosa*, two major siderophore molecules, pyochelin and pyoverdine, have been extensively studied to determine their roles in iron metabolism^15^,^37^,^38^. As was shown in our microarray results, expression of genes involved in the production of these two molecules was also upregulated in response to treatment with AMS. We therefore sought to examine the relative importance of each system in terms of responsiveness to AMS. To address this issue, we constructed a series of mutants and calculated the growth index of each mutant. *P. aeruginosa* single mutants deficient in the production of either pyochelin or pyoverdine were completely viable in response to AMS treatment (Fig. 4A). Importantly, a Δ*pvd*Δ*pch* double mutant defective in both pyochelin and pyoverdine also remained viable in response to the same treatment. Of note, the growth indices of the Δ*pvd*, Δ*pch*, or Δ*pvd*Δ*pch* mutants were almost identical to that of PAO1, suggesting that the ability to produce these two siderophores is not required for the protective response to AMS. In contrast, when the *PA4834* gene was additionally disrupted, all the mutants (Δ*pch*Δ*PA4834*, Δ*pvd*Δ*PA4834* and Δ*pvd*Δ*pch*Δ*PA4834*) were susceptible to the antimicrobial action of AMS (Fig. 4A).

We next examined whether the susceptible Δ*PA4834* mutant could be rescued from the AMS treatment by supplementation with bacterial culture supernatants. As shown in Fig. 4B, when the Δ*PA4834* mutant was grown in AMS amended with cell-free culture supernatants from the Δ*pvd*Δ*pch* mutant, its growth index was significantly increased. Culture supernatant of the triple mutant, however, failed to rescue the Δ*PA4834* mutant (Fig. 4B) and the growth index was quite similar to that of the mutant grown in AMS. This result further demonstrates that secretory molecule(s), the production of which is dependent on the presence of the *PA4834* gene product, is critically necessary for the survival and proliferation of *P. aeruginosa* in AMS.

### Supplementation of nicotianamine (NA) restores the ability of the *ΔPA4834* mutant to grow in AMS

**Figure 5:**
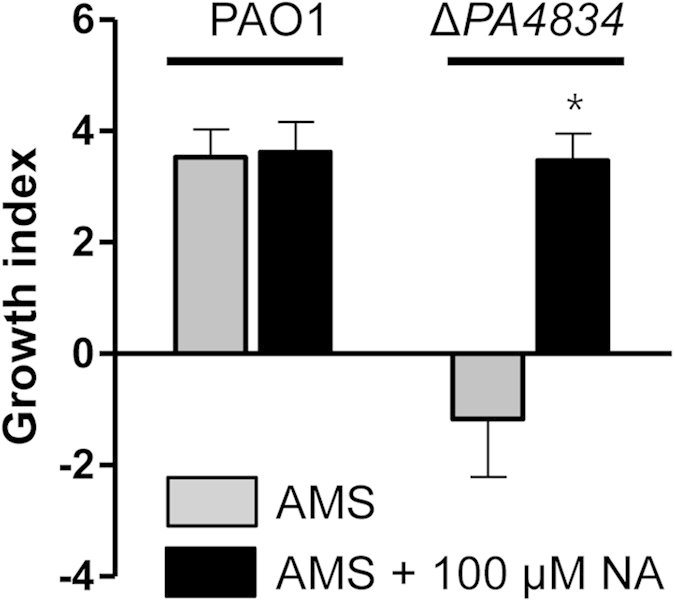
Effect of the addition of nicotianamine (NA) on Δ*PA4834* mutant growth in AMS. PAO1 and the Δ*PA4834* mutant were grown in AMS (gray bars) and AMS amended with 100 μM NA (black bars) and growth indices were measured. Experimental conditions were identical to those described in Fig. 1C. Three independent experiments were performed, and mean values ± SD (error bars) are displayed in each bar. *p < 0.05 vs. growth of the Δ*PA4834* mutant in AMS with no NA supplementation, as determined by students’ t-test.

The *PA4836* gene encodes a putative NA synthase. NA is a compound with high-affinity for iron and it has been extensively studied in plants^39^,^40^. Furthermore, the *PA4837* gene product likely acts as a siderophore receptor suggesting the involvement of this genetic element in iron acquisition. This information led us to postulate that *P. aeruginosa* may also produce NA to sustain its growth in AMS. To address this issue, we examined whether NA supplementation could rescue growth of the Δ*PA4834* mutant in AMS. Similar to previous results, PAO1 was capable of growing in AMS regardless of the presence of 100 μM NA (Fig. 5). Importantly, growth of the Δ*PA4834* mutant was completely restored by the presence of 100 μM NA (Fig. 5).

### *In vivo* virulence of the *ΔPA4834* mutant is attenuated

**Figure 6:**
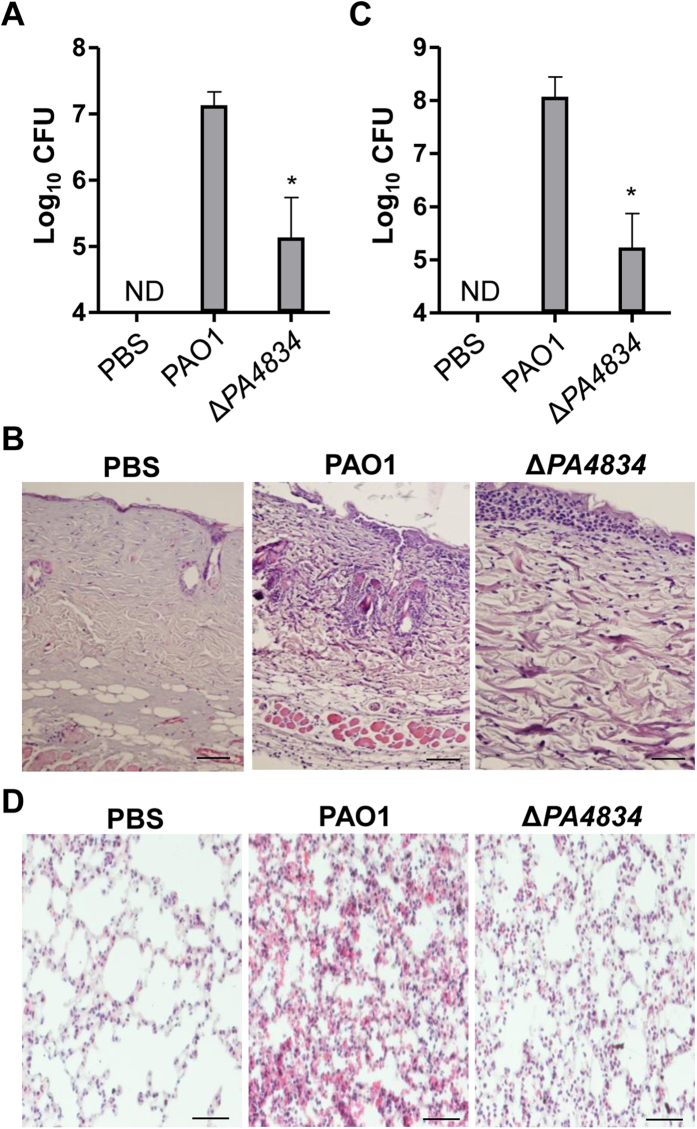
*In vivo* infectivity of the Δ*PA4834* mutant is decreased. (**A,B**) Mouse burn wound infection. (**A**) PAO1 and the Δ*PA4834* mutant were infected in the thermally-injured region of the mouse skin. The infection dose was 2 × 108 bacterial cells. After 24 h, the infected area was incised and homogenized to enumerate viable cells. Three independent experiments were performed, and mean values ± SD (error bars) are displayed in each bar. *p < 0.05 vs. CFU of PAO1, as determined by students’ t-test. ND, not detected. (**B**) Histological images of infected skin sections of mice challenged with PBS (negative control), PAO1, or the Δ*PA4834* mutant. (**C,D**) Mouse airway infection. (**C**) PAO1 and the Δ*PA4834* mutant (1 × 10^7^ CFU) were exposed to the mouse airway via the intranasal route. After 24 h, mouse lungs were removed and homogenized to enumerate viable cells. Three independent experiments were performed, and mean values ± SD (error bars) are displayed in each bar. *p < 0.05 vs. CFU of PAO1, as determined by students’ t-test. ND, not detected. (**D**) Representative histological images of lung sections of mice challenged with PBS (negative control), PAO1, or the Δ*PA4834* mutant.

We next examined whether *in vivo* infectivity of PAO1 was affected by deletion of the *PA4834* gene by employing the burn wound infection and airway infection mouse models. In both cases, infectivity of the Δ*PA4834* mutant was significantly compromised. The average number of viable cells recovered from the infected burn wound areas indicated that the mutant was less capable of proliferating than its parental strain PAO1 (Fig. 6A). Furthermore, while severe inflammation was clearly induced by infection with PAO1, the degree of inflammation was considerably decreased by infection with the same dose of the Δ*PA4834* mutant (Fig. 6B). The attenuation of bacterial virulence by the *PA4834* gene deletion was also dramatic in the acute mouse lung infection model. The average number of viable cells enumerated from the lung of PAO1-infected mice was ~1.2 × 108/ml, while it was decreased to ~1.7 × 105/ml in mice initially infected with the same number of mutant cells (Fig. 6C), clearly suggesting that the mutant was not competent in establishing airway infection. As expected, a high degree of inflammation was only induced in the lung tissues of mice infected with PAO1, but not with the Δ*PA4834* mutant (Fig. 6D).

## Discussion

Airway mucus is a key component of the innate immunity that mediates the elimination of inhaled microorganisms via mucociliary clearance^27^,^41^,^42^. It contains antimicrobial components such as lysozymes and defensin^43^. The bactericidal activity, however, is compromised in the diseased CF airway due to the abnormally altered mucus environment and *P. aeruginosa* is capable of establishing recalcitrant infection under such conditions^4^,^9^,^44^. Given the fact that the CF patient airway is equally exposed to various opportunistic pathogens, it remains a question as to why *P. aeruginosa* has been so successful in colonizing and proliferating in the CF patient airway^11^,^45^,^46^. Here, we addressed this long-standing question using *ex vivo* AMS experimental conditions to study the host-microbe interaction, mimicking the early stages of airway infection. *P. aeruginosa* was capable of propagating upon interaction with AMS, a finding not observed in other tested bacterial species. These data strongly suggest that *P. aeruginosa* infection in patients with CF, COPD, or ventilator care stems from the specific response of *P. aeruginosa* to the airway environment and not from the abnormal composition of the airway mucus *per se*. The growth of *P. aeruginosa* in nasal secretions collected from healthy volunteers has been previously reported^47^; since then, it has been further elucidated that airway mucus provides the carbon source *P. aeruginosa* needs to proliferate^45^,^48^.

We then explored mechanisms by which *P. aeruginosa* is resistant to airway mucus, which is a completely different response than what is seen in other bacterial species. We focused on the PAO1 genes upregulated when exposed to AMS. Our microarray data showed that various genes related to iron uptake and metabolism were upregulated when *P. aeruginosa* is exposed to AMS. In particular, genes that encode proteins involved in the production of siderophores such as pyoverdine and pyochelin were substantially upregulated, which is remarkably similar to previous studies where *P. aeruginosa* was exposed to airway mucus and low iron environments^49^,^50^,^51^. *P. aeruginosa* is known to have a special ability to utilize small amounts of iron by the synthesis of siderophores such as pyoverdine and pyochelin^15^,^52^,^53^,^54^. We also confirmed that PAO1 has higher siderophore activity than other bacteria. Based on these findings, we assumed that the selective resistance of *P. aeruginosa* to airway mucus came from the ability of *P. aeruginosa* to survive in low iron concentration conditions via a special iron-utilizing system.

To test this assumption, we screened for the critical gene(s) necessary for the survival of *P. aeruginosa* in airway mucus using transposon insertion mutants with special attention to genes regulating iron metabolism. In contrast to our expectations, transposon mutants defective in the production of pyoverdine and pyochelin, chemicals known to be important for iron acquisition, were able to survive upon treatment with AMS, while the *PA4834* mutant lost its resistance to airway mucus. The *PA4834* gene encodes a membrane-associated protein (284 amino acids) that contains a duplication of the evolutionarily conserved EamA domain, a well-characterized motif present in proteins belonging to the Drug and Metabolite Transporter (DMT) family. Of note, PA4834 has a 51% amino acid similarity to the S-adenosylmethionine (SAM) uptake transporter of *Paracoccus aminophilus* strain JCM 7686. In addition, SAM is a precursor of NA, a well-characterized phytosiderophore produced by plants^55^,^56^. The *PA4834* gene is part of a putative operon with the *PA4835*, *PA4936* and *PA4837* genes, and a BLASTN search demonstrated that this particular gene cluster is exclusively present in *P. aeruginosa* strains. The *PA4837* gene is predicted to encode a TonB-dependent siderophore receptor. The *PA4836* gene, although annotated as a hypothetical protein in the PAO1 genome database, likely encodes a protein homologous to NA synthase. Therefore, it was reasonable to hypothesize that this operon is related to a new iron transport system and the possession of this system may account for the resistance of *P. aeruginosa* to airway mucus. Although the primary sequence of the PA4834 protein is similar to the SAM uptake transporter, the possibility of PA4834 acting as a SAM transporter is low because the concentration of intracellular SAM was comparable in both the PAO1 and Δ*PA4834* mutant (data not shown). Importantly, the ability of the Δ*PA4834* mutant to grow in AMS was recovered by the extraneous addition of NA. Thus, it is highly possible that PA4834 encodes a protein involved in NA secretion. Consistent with our finding, previous genome-wide studies also reported the potential importance of this genetic region for the survival of *P. aeruginosa* in the airway environment. First, the *PA4834*-*PA4837* operon has been shown to be upregulated in *P. aeruginosa* strain PA14 growing on artificial sputum medium as a sole carbon source^45^. Second, the *PA4837* gene was found to be expressed in *P. aeruginosa* clinical CF isolates early in CF infection^57^. Third, expression of genes in this operon was also markedly increased when *P. aeruginosa* strain PA14 was grown in the peritoneal cavity of a rat^58^. Lastly, the *PA4935* and *PA4836* genes were exclusively expressed under in *in vivo* burn wound infections^59^. These findings, together with our results, strongly suggest that the *PA4834*-*PA4837* operon plays an essential role in bacterial proliferation in airway mucus. Under iron-replete conditions like LB media, both PAO1 and the Δ*PA4834* mutant may sequester iron with siderophores such as pyochelin and pyoverdine. However, our results demonstrate that bacterial growth of the PAO1 mutant defective in production of these two molecules alone was not affected during incubation with AMS, while the *PA4834* gene mutation resulted in a significant defect in bacterial ability to cope with AMS-induced stress.

Although this study identified a new genetic element that accounts for the exceptional ability of *P. aeruginosa* to propagate in human airway mucus, our study does have the following limitations. First, because AMS samples were only provided in small quantities, we were only able to grow *P. aeruginosa* strains for a short period of time. Considering that *P. aeruginosa* establishes chronic biofilm infection in the CF airway^42^, our experiments using AMS may not be completely relevant to describe *P. aeruginosa* pathogenesis *in vivo*. Second, because AMS samples were prepared from outpatients with varying degrees of health conditions, differential antimicrobial activities were observed among the AMS preparations. Regardless, PAO1 cells were always resistant to AMS treatment while the Δ*PA4834* mutant exhibited susceptibility to the same treatment. These difficulties could be overcome by constructing a stable airway cell line that secretes mucus with predictable composition. Lastly, deletion of the *PA4834* gene resulted in a dramatic phenotype in response to AMS incubation and the sole addition of NA completely rescued the ability of the Δ*PA4834* mutant to grow in AMS. However, more experiments are necessary to precisely understand the molecular basis of how NA is produced, secreted, and internalized to act as a siderophore and how each gene product of the operon contributes to this process.

This study shows the robust capability of *P. aeruginosa* to survive and propagate inside the AMS that contains various antimicrobial components. The AMS samples used here were collected from primarily cultured normal human tracheal epithelial (NHTE) cells, and this fact may suggest that *P. aeruginosa* can also colonize the normal airway. However, *P. aeruginosa* airway infection in healthy individuals is not prevalent, suggesting that physical mucociliary clearance and a functional immune system play more important roles than antimicrobial agents inside the AMS in preventing *P. aeruginosa* infection. Provided that the AMS composition is similar between normal and CF airways, frequent *P. aeruginosa* infection in CF airways is therefore attributed more to the disease-associated symptoms, such as impaired mucociliary clearance and ineffective neutrophil-mediated immune attack.

In conclusion, our data show that *P. aeruginosa* can selectively grow in airway mucus by utilizing a newly identified iron uptake system. Our present results provide fundamental insight into the competent behavior of *P. aeruginosa* under airway-specific environments. Selective growth of *P. aeruginosa* in the CF airway is caused by the ability of this important pathogen to manage adverse mucus conditions, such as low iron availability, and not due to the compositional changes of the airway mucus *per se*. Therefore, any condition where the mucociliary clearance system is impaired in patients with CF, COPD, or prolonged ventilator use can lead to *P. aeruginosa* infection. Our findings also suggest that the suppression of *PA4834* gene expression or blocking the biological activity of the *PA4834* gene product can be novel approaches to the treatment of *P. aeruginosa* infections.

## Methods

### Experimental ethics

Experiments using human subjects and experimental animals were performed in strict accordance with guidelines provided by Yonsei University. Protocols were reviewed and approved by Institutional Review Board of Yonsei University College of Medicine. Permit numbers for primary culture of human tissues and mouse infection experiment were 2014-1842-001 and 2011-0173-2, respectively.

### Bacterial strains, cell lines, and growth conditions

Bacterial strains and plasmids used in the current study are listed in Table S1. Luria-Bertani medium (LB; 10 g tryptone, 5 g yeast extract, and 10 g NaCl per liter) was used to grow bacterial precultures. For bacterial treatment in AMS, bacterial cultures grown in LB for 8 h were diluted in PBS to get bacterial suspensions with the CFU of 106/mL. Dilution ratios for each bacterial species were pre-determined based on the CFU of 8 hr cultures. Ten μl of each diluent was inoculated into 100 μL AMS to achieve the initial inoculum size of ~105 CFU/mL in AMS. Bacterial cells were then grown for 16 h in a humidified 37 °C incubator. Bacterial growth was assessed by measuring the growth index. Unless otherwise noted, the values for log_10_ (CFU_after 16 h in AMS or LB_/CFU_after 16 h in PBS_) were calculated and plotted as the growth index. Transposon insertion mutants of *P. aeruginosa* were purchased from a *P. aeruginosa* transposon mutant library (http://www.genome.washington.edu/UWGC/pseudomonas). The targeted gene mutations were introduced by allele replacement as previously described^11^.

### Preparation of airway mucus secretions (AMS)

AMS was collected from primary cultures of normal human tracheal epithelial (NHTE) cells following procedures described previously^60^. In brief, a piece of human trachea was harvested from patients without airway disease during tracheotomy. Second passage NHTE cells (105 cells/culture) were seeded in 0.5 ml of culture medium onto 24.5 mm, 0.45 mm pore size Transwell^®^ clear culture inserts (Costar Co., Cambridge, MA). Cultured cells were grown submerged, and the culture medium was changed on the first day and every other day thereafter. After cultures reached confluence, media on the luminal side was removed to create an air–liquid interface (ALI) to facilitate differentiation into ciliated columnar epithelial cells with polarity. To harvest AMS, we washed the apical surface of the NHTE cells with 1 ml PBS. All patients provided informed consent, and the Institutional Review Board of Yonsei University College of Medicine approved this study (2014-1842-001). Protein components of the AMS samples were analyzed by SDS-PAGE coupled with Mass Spectrometry^61^. The presence of the Muc5AC protein was verified by dot-blot analysis using an anti-Muc5AC antibody^62^.

### Complementation of the *ΔPA4834* mutant

A 0.86-kb NdeI-HindIII fragment containing the entire *PA4834* gene was amplified from the PAO1 genome and ligated into NdeI/HindIII-treated pET21b. To complement the Δ*PA4834* mutant, the arabinose-inducible expression vector pJN105^63^ was used. PCR was used to amplify the *PA4834* gene along with the ribosome binding site (RBS) that originated from pET21b. The EcoRI/XbaI-digested PCR product was ligated into EcoRI/XbaI-digested pJN105. The resulting plasmid and the control empty plasmid (i.e., pJN105) were transferred into the Δ*PA4834* mutant by electroporation.

### Microarray analysis

Genome-wide transcriptome analysis was performed using GeneChip *P. aeruginosa* genome arrays (Affymetrix, Santa Clara, CA). PBS-washed PAO1 cells precultured in LB overnight were incubated for 6 h in PBS or in AMS. RNA extraction, labeling, hybridization, and data analysis were performed as described elsewhere^11^.

### Antibiotic sensitivity assay

Commercially purchased filter discs (BBL Sensi-Disc susceptibility test discs; Becton, Dickinson and Company, Franklin Lakes, NJ) were used. After overnight incubation of PAO1, and Δ*PA4834* mutant cells on LB agar plates containing various bacteria the zones of inhibition were measured.

### Quantitative real time PCR

*PA4834*, *PA4835*, *PA4836*, and *PA4837* gene transcript levels were determined by qRT-PCR, following previously described procedures^11^. Prior to RNA extraction, PAO1 cells were grown for 6 h in 1 mL of LB or AMS with shaking at 37 °C. Transcript levels of the *rpoD* gene were also measured and used for normalizing *PA4834*, *PA4835*, *PA4836*, and *PA4837* gene transcription. The primers used for qRT-PCR are listed in Table S2.

### Scanning electron microscope

Primary cultured NHTE cells grown on permeable membrane (Costar Co., Cambridge, MA) were inoculated with overnight cultured PAO1, SA or Δ*PA4834* mutant (2 × 10^4^ CFU in 300 μl PBS). After 14 h incubation, the NHTE cells were fixed, dried and coated with platinum using a sputter coater (E1030; Hitachi).Coated specimens were then mounted on a stub holder and viewed using a cold-field emission scanning electron microscope (S-4300, Hitachi) operated at 15 kV.

### Intracellular iron concentration

Inductively coupled plasma mass spectrometry (ICP-MS) was performed to determine the intracellular concentration of total iron. Bacterial pellets grown in LB or AMS were submitted to the Korea Basic Science Institute (Seoul, Korea), where subsequent procedures were performed. The cell-permeable green fluorescent heavy metal indicator, PhenGreen™ SK, diacetate (Molecular Probes, Inc., Eugene, OR) was used to fluorescently stain bacterial cells. Aliquots of bacterial suspensions were stained with 20 μM PhenGreen™ SK, diacetate for 30 min and fluorescent images were acquired using a fluorescent microscope (Zeiss LSM 700; Carl Zeiss Inc., Germany).

### Iron sequestration assay

Bacterial strains were grown in LB and cell-free culture supernatants were subject to the iron sequestration assay using a colorimetric iron assay kit according to the manufacturer’s instructions (SideroTech Kit. Emergen Bio Inc., Ireland). The assay is based on the color change that occurs as result of ferric iron transfer from the reagent complex to siderophore present in culture supernatants.

### Mouse infection

Methods for animal infection were approved by the Yonsei University College of Medicine Committee on the Ethics of Animal Experiments (permit number 2011-0173-2). Effects of the *PA4834* gene deletion on *P. aeruginosa in vivo* infectivity was monitored using two different mouse infection models, the thermally-injured mouse infection model^64^ and the acute airway infection^65^ model. For thermal injury, adult male C57BL/6 mice were anaesthetized and their backs were shaved. Thermal injury was induced by exposing the shaved skin (15% of their body surface) to an aluminum plate (16 mm diameter) heated to 99 °C for 8 sec. Immediately after, sterilized gauze was fixed on the injured skin and the mice were then challenged by spraying 0.2 ml of 2 × 10^8^ *P. aeruginosa* CFU or 0.2 ml PBS (as the control) on the gauze. Acute airway infection was performed following the procedures described previously^65^. For both infection experiments, affected tissues were recovered and stained with hematoxylin-eosin for histological analysis^65^.

### Statistical analysis

Data are expressed as the mean ± standard deviation (SD). Unpaired Student’s *t-*test and ANOVA were used to analyze the data. A *p* value of <0.05 was considered statistically significant. All the experiments were repeated for reproducibility.

## Additional Information

**How to cite this article**: Gi, M. *et al.* A novel siderophore system is essential for the growth of *Pseudomonas aeruginosa* in airway mucus. *Sci. Rep.* **5**, 14644; doi: 10.1038/srep14644 (2015).

## Supplementary Material

Supplementary Information

Supplementary Dataset 1

## Figures and Tables

**Table 1:** List of genes that are most upregulated during growth with AMS.

PA number	Gene	Fold change^a^	Target Description
PA3446	—	56.63	conserved hypothetical protein
PA3600	—	49.03	conserved hypothetical protein/Translation, post-translational modification
PA0284	—	36.28	hypothetical protein
PA4063	—	35.15	hypothetical protein
PA3601	—	24.27	conserved hypothetical protein/Translation, post-translational modification
PA2359	—	21.93	probable transcriptional regulator
PA3450	—	21.63	probable antioxidant protein/adaptation, protection
PA4570	—	19.36	hypothetical protein
PA4471	—	18.45	hypothetical protein
PA2161	—	16.99	hypothetical protein
PA0802	—	16.82	hypothetical protein/Membrane proteins
PA5536	*dksA2*	15.69	DnaK suppressor protein
PA2062	—	15.52	probable pyridoxal-phosphate dependent enzyme/Putative enzymes
PA1911	*femR*	13.79	sigma factor regulator
PA2033	—	11.83	hypothetical protein
PA1905	*phzG2*	11.29	probable pyridoxamine 5′-phosphate oxidase/Secreted Factors
PA4230	*pchB*	10.98	salicylate biosynthesis protein PchB/Transport of small molecules; Secreted Factors
PA1373	*fabF2*	10.86	3-oxoacyl-acyl carrier protein synthase II/Fatty acid and phospholipid metabolism
PA4103	—	10.61	hypothetical protein
PA4224	*pchG*	10.61	pyochelin biosynthetic protein PchG
PA2570	*lecA*	10.15	PA-I galactophilic lectin/Adaptation, protection; Motility & Attachment Cell wall
PA2786	—	9.98	hypothetical protein
PA1300	—	9.66	probable sigma-70 factor, ECF subfamily/Transcriptional regulators
PA3381	—	9.55	probable transcriptional regulator/Transcriptional regulators
PA0993	*cupC2*	9.36	chaperone CupC2/probable pili assembly chaperone/Motility & Attachment
PA4033	*mucE*	9.01	hypothetical protein
PA4837	—	8.95	probable outer membrane protein precursor/Membrane proteins
PA4516	—	8.93	hypothetical protein
PA4834	—	8.33	hypothetical protein/Membrane proteins
PA4226	*pchE*	8.27	dihydroaeruginoic acid synthetase/Transport of small molecules; Secreted Factors
PA0790	—	8.05	hypothetical protein/Membrane proteins
PA3126	*ibpA*	7.92	heat-shock protein LbpA/Chaperones & heat shock proteins
PA1312	—	7.84	probable transcriptional regulator/Transcriptional regulators
PA2426	*pvdS*	7.76	sigma factor PvdS/Transcriptional regulators
PA4225	*pchF*	7.23	pyochelin synthetase/Transport of small molecules; Secreted Factors

^a^Fold change was based on expression level in PAO1 incubated in PBS for the same period of time.

**Table 2:** Effects of the disruption of upregulated genes on sensitivity to AMS treatment.

Strains	Growth index	Strains	Growth index
PAO1	2.46 (±0.61)	PA1373::Tn	1.48
PA3446::Tn	2.23	PA4103::Tn	1.75
PA3600::Tn	1.71	PA4224::Tn	1.16
PA0284::Tn	1.77	PA2570::Tn	1.34
PA4063::Tn	−0.06	PA2786::Tn	not tested
PA3601::Tn	1.92	PA1300::Tn	1.45
PA2359::Tn	1.54	PA3381::Tn	1.03
PA3450::Tn	not tested	PA0993::Tn	1.45
PA4570::Tn	1.58	PA4033::Tn	2.20
PA4471::Tn	1.18	PA4837::Tn	1.93
PA2161::Tn	1.54	PA4516::Tn	1.90
PA0802::Tn	1.85	PA4834::Tn	−0.18
PA5536::Tn	2.31	PA4226::Tn	2.94
PA2062::Tn	1.54	PA0790::Tn	2.34
PA1911::Tn	1.32	PA3126::Tn	3.56
PA2033::Tn	not tested	PA1312::Tn	3.79
PA1905::Tn	1.53	PA2426::Tn	3.50
PA4230::Tn	not tested	PA4225::Tn	4.00
