# Supplementary material for: A novel siderophore system is essential for the growth of Pseudomonas aeruginosa in airway mucus
Source: Sci Rep. 2015 Oct 8;5:14644. doi: 10.1038/srep14644 (PMC4597187; doi:10.1038/srep14644)
Supplement: Supplementary Information [file srep14644-s1.pdf]

Supplementary Information

A novel siderophore system is essential for the growth of *Pseudomonas aeruginosa* in airway mucus

Mia Gi[a#], Kang-Mu Lee[b#], Sang Cheol Kim[a], Joo-Heon Yoon[a], Sang Sun Yoon[b,c*], and Jae Young Choi[a*]

Table S1. Bacterial strains and plasmids used in this study

Strains or plasmids	Relevant characteristics	Ref. or source
P. aeruginosa strains		
PAO1	Wild-type PAO1, ATCC	Lab collection
Δ*PA4834*	PA01, *PA4834* deleted	This study
Δ*pvd*	PAO1, *PA2396* and *PA2397* deleted	This study
Δ*pch*	PAO1, *PA4224* and *PA4225* deleted	This study
Δ*pvd*Δ*pch*	PAO1, *PA2396, PA2397, PA4224* and *PA4225* deleted	This study
Δ*PA4834*Δ*pvd*	PA01, *PA4834, PA2396* and *PA2397* deleted	This study
Δ*PA4834*Δ*pch*	PA01, *PA4834, PA4224* and *PA4225* deleted	This study
Δ*PA4834*Δ*pvd*Δ*pch*	PA01, *PA4834, PA2396, PA2397, PA4224* and *PA4225* deleted	This study
Δ*PA4834*::Tn	*PW9128, PA4834*:: ISlacZ/hah, UW strain	[60]
Δ*PA4834*/pJN105	Δ*PA4834*, empty vector control	This study
Δ*PA4834*/pJN105::*PA4834*	Δ*PA4834* complementation strain	This study
E. coli strains		
SM10/λpir	Km^r *thi-1 thr leu tonA lacY supE recA*::RP4–2-Tc::Mu pir^+, for conjugal transfer	[61]
Other bacterial species		
Staphylococcus aureus		Lab collection
Bacillus cereus		Lab collection
Escherichia coli	DH5α	Lab collection
Vibrio cholerae	N16961	Lab collection
Salmonella enterica serovar Typhimurium	LT2	Lab collection
Listeria monocytogenes		Lab collection
Plasmids		
pCVD442	*sacB* suicide vector from plasmid pUM24	[61]
pJN105	pJN105 *araC*-PBAD cassette cloned in pBBR1MCS-5, Gm^r	[57]
pJN105::*PA4834*	pJN105 inducible vector with fragment of *PA4834*	This study

Table S2. Primers used in this study

Gene name	Direction	Primer sequence (5'-3')[a]	Restriction sites
Mutant construction			
PA4834 left	Forward	ATATAGAGCTCGCCGAGCGCCAGGAATACCTG	SacI
PA4834 left	Reverse	TAAATGGATCCCAGCAGGGTACCGATGGCGC	BamHI
PA4834 right	Forward	CCCTTGGATCCGGCATCGTCTTCAACGCCGG	BamHI
PA4834 right	Reverse	AAGGTGAGCTCACGCCTTTCTGCCTGGTCACG	SacI
pvd left	Forward	ACACGGAGCTCCCCTTCCCGTTCGACGAAGGTA	SacI
pvd left	Reverse	CTTCCGGATCCTTCACCCGACGACACCATCCTC	BamHI
pvd right	Forward	AGAGCGGATCCAATGCCTGGCTCGAAGAGCGAC	BamHI
pvd right	Reverse	CGATTGAGCTCACTCCTGGAGTGCGCTGCCC	SacI
pch left	Forward	ACACGGAGCTCAATGGCAAGGTCGATCGTCGC	SacI
Pch left	Reverse	CTTCCGGATCCCAGGTGTTCCAGCAGGGCCTC	BamHI
pch right	Forward	AGAGCGGATCCAACCAGCGCAGCCTGGAGGT	BamHI
pch right	Reverse	CGATTGAGCTCAGCGTGCGCGATCAGTTGGT	SacI
complementation			
PA4834 complementation (cloning to pET21b)	Forward	CTTACCATATGGTGCTCGACCTGCTGAAGAGC	NdeI
	Reverse	AAATTAAGCTTGCCCTTCTTCGTCCGCGACAG	HindIII
PA4834 complementation (cloning to pJN105)	Forward	ACTCTGAATTCAATAATTTTGTTTAACTTTAAGAAGGAG	EcoRI
	Reverse	ATTGGTCTAGATCAGTGGTGGTGGTGGTG	XbaI
Quantitative RT-PCR			
PA4834	Forward	GACCTGCTGAAGAGCGGGGT	
	Reverse	GCTTCCTTGACCAGGGCGTT	
PA4835	Forward	TACGTGCACAGTCCGTTCTT	
	Reverse	GATAGGTCCCTCCGGGTAGA	
PA4836	Forward	CTGTTCAACTATCCGGTGGAG	
	Reverse	TTCTCCAGGATCAGGGTGTC	
PA4837	Forward	ACCTTGACAGCGCTCGTATC	
	Reverse	AAGCCACGGACGTTGTACTC	
rpoD (PA0576)	Forward	AAGGCCCTGAAGAAGCACGG	
	Reverse	GATCGGCATGAACAGCTCGG	

[a] Restriction enzyme recognition sequences are underlined.

Table S3. BLASTN search of *PA4834*, *PA4835*, *PA4836* and *PA4837* gene sequences

Genes	**PA4834**	**PA4835**	**PA4836**	**PA4837**
Predicted function	EamA-like transporter family protein	Conserved uncharacterized	Nicotianamine synthase	TonB-dependent siderophore receptor
**Bacterial species that contain genes homologous to *PA4834*, *PA4835*, *PA4836* or *PA4837***				
P. aeruginosa PA01	855 / 855 (100%)	1302 / 1302 (100%)	792 / 792 (100%)	2127 / 2127 (100%)
P. aeruginosa DK2	855 / 855 (100%)	1296 / 1302 (99%)	787 / 792 (99%)	2123 / 2127 (99%)
P. aeruginosa LESB58	853 / 855 (99%)	1295 / 1302 (99%)	784 / 792 (99%)	2114 / 2127 (99%)
P. aeruginosa M18	853 / 855 (99%)	1296 / 1302 (99%)	786 / 792 (99%)	2120 / 2127 (99%)
P. aeruginosa RP73	853 / 855 (99%)	1297 / 1302 (99%)	786 / 792 (99%)	2118 / 2127 (99%)
P. aeruginosa PA96	854 / 855 (99%)	1299 / 1302 (99%)	785 / 792 (99%)	2115 / 2127 (99%)
P. aeruginosa UCBPP-PA14	846 / 854 (99%)	1286 / 1302 (99%)	783 / 792 (99%)	2108 / 2127 (99%)
P. aeruginosa PA7	788 / 855 (92%)	1188 / 1302 (91%)	757 / 792 (96%)	2001 / 2127 (94%)
P. protegens Cab57	–	–	–	1436 / 2007 (72%)
P. stutzeri A1501	–	–	–	1465 / 2062 (71%)
P. mendocina ymp	–	–	–	1478 / 2094 (71%)
P. mandelii JR-1	–	–	–	1432 / 2017 (71%)
Burkholderia sp. 383	577 / 828 (70%)	–	–	–
Serratia sp. FS14	530 / 782 (68%)	255 / 373 (68%)	–	–
Serratia marcescens WW4	514 / 759 (68%)	–	–	–
Arsenophonus nasoniae	487 / 726 (67%)	–	–	–
Vibrio nigripulchritudo str. SFn1	250 / 385 (65%)	–	–	–
Actinosynnema mirum DSM 43827	–	207 / 285 (73%)	–	–
Bordetella bronchiseptica 253	–	–	38 / 43 (88%)	–
Bordetella bronchiseptica MO149	–	–	38 / 43 (88%)	–
Azotobacter vinelandii CA6	–	–	–	1480 / 2023 (73%)
Azotobacter vinelandii CA	–	–	–	1480 / 2023 (73%)

Table S4. Antibiotic sensitivity test

Antibiotics	Inhibition zone (mm)	
	PA01	Δ*PA4834*
Amikacin	24	26
Ceftazidime	27	29
Ciprofloxacin	34	33
Gentamicin	19	21
Tobramycin	26	26
Cefoperazone-sulbactam	27	27
Piperacillin	28	28
Imipenem	24	26
Piperacillin-tazobactam	29	30
Aztreonam	26	27
Meropenem	31	31
Cefepime	30	31
Colistin	13	13
